# Different Drought-Tolerant Mechanisms in Quinoa (*Chenopodium quinoa* Willd.) and Djulis (*Chenopodium formosanum* Koidz.) Based on Physiological Analysis

**DOI:** 10.3390/plants10112279

**Published:** 2021-10-24

**Authors:** Pin-Hua Lin, Yun-Yang Chao

**Affiliations:** Department of Plant Industry, National Pingtung University of Science and Technology, No. 1, Shuefu Road, Neipu, Pingtung 912301, Taiwan; Srps55664@gmail.com

**Keywords:** drought tolerance mechanism, antioxidant capacity, oxidative stress, osmotic regulation, yield

## Abstract

The purpose of this experiment is to study the effects of treatment with 90% (28.5% volumetric water content (VWC)), 75% (24% VWC), 50% (16% VWC), and 25% (8% VWC) of water requirements on the growth of two djulis (*Chenopodium formosana* Koidz) varieties (red: RP and yellow: OR) and one quinoa (*Chenopodium quinoa* Willd) varieties (PI). The results showed that drought stress (8% VWC) significantly reduced plant growth and relative water content, and increased H_2_O_2_ and MDA content in *C. formosana* and *C. quinoa*. The most significant increase in these parameters was detected in the OR variety. The antioxidant enzymes, such as SOD, APX, and GR activities of PI variety under drought treatment (8% VWC), are significantly increased, while GR activity of *C. formosana* also increased significantly. Additionally, *C. formosana* and PI variety remained at a stable AsA/DHA ratio, but the GSH/GSSG ratio decreased during drought treatment. Moreover, drought stress increased total soluble sugars and proline content in the PI variety. However, *C. formosana* proline content was extremely significantly enhanced, and only the OR variety increased the total soluble sugar content at the same time during the vegetative growth period. In summary, *C. formosana* and *C. quinoa* have different drought tolerance mechanisms to adapt to being cultivated and produced under severe drought conditions.

## 1. Introduction

Global precipitation intensities and temperature fluctuations are more severe as a result of extreme weather conditions. In many regions, crop cultivation is rendered impossible due to the lack of water resources, indicating that drought stress has significantly impacted crop production. Therefore, issues such as insufficient food production in the future and global food security have become major concerns [1,2]. Under drought stress, water absorption by crops decreases, which causes a decrease in the water potential of cells and induces osmotic stress. The decreased water potential changes cell turgor pressure and impedes cell elongation. Furthermore, it decreases stomatal conductance and reduces CO_2_ supply, photosynthetic rate, leaf areas, and plant height [3,4]. The low photosynthetic rate due to insufficient water supply may indirectly affect carbohydrate metabolism and inhibit the biosynthesis of sucrose, which in turn affects the rate of sucrose transportation to the reproductive organs and eventually causes a low grain-filling rate, affecting the crop yield [5]. Moreover, drought stress may induce the generation of reactive oxygen species (ROS) in the crops, which damages lipid and protein structures, causing the cell membrane to lose permeability and selectivity. The resulting leakage of intracellular ions leads to a disturbance of metabolism, disintegration of chloroplast, and lowering of chlorophyll content. In severe cases, it may lead to plant death [6].

Under drought stress, the crops may accumulate osmosis-regulating substances, which would stabilize cell osmotic potential and maintain cell turgor pressure and relative water content while maintaining stomatal conductance and photosynthesis. Such substances include soluble sugars (trehalose, sucrose, glucose, and fructose), polyols (mannitol, inositol, and sorbitol), amino acids (trimethylglycine, proline, etc.), and polyamines [3,4,7]. In addition, the crops can also rely on a built-in antioxidative system to eliminate the oxidative stress induced by accumulated ROS under drought stress. The system may harness important antioxidant enzymes, such as superoxide dismutase (SOD), catalase (CAT), ascorbate peroxidase (APX), and glutathione reductase (GR) in addition to antioxidants, such as glutathione (GSH) and ascorbate (AsA), to alleviate the oxidative stress induced by droughts [4,8].

Quinoa (*Chenopodium quinoa* Willd.) is a member of the family Amaranthaceae. The plant originated in the Andean region of South America. It is a traditional local crop with extraordinary tolerance against abiotic stresses, such as frost, salinity, and drought [9]. Quinoa can grow in dry environments with annual precipitation of at least 200 mm. Research has revealed that quinoa can withstand drought stress by increasing water absorption to promote root growth and raising proline and total soluble sugar content to regulate cell osmotic potential [10,11]. The quinoa PI478414 variety is derived from La Paz (Bolivia) and grows at an altitude of 3800 m. The grain is dark in color and highly resistant to downy mildew. Bhargava et al. (2006) [12] applied for 27 germplasm lines from India’s National Botanical Research Institute for yield experiments. Two years of test results show that the grain yield of the PI478414 variety is 6083 kg per hectare (ranking 7th), hence showing that PI478414 variety has better drought tolerance. Bhargava et al. (2008) [13] selected 29 varieties (including PI478414 variety) to explore the relationship between appearance traits and yield and quality of quinoa. Morphological traits, including plant height, leaf size, stem diameter, ear length, ear number, thousand-grain species, and grain weight are used as the criteria. Those investigated traits have a positive correlation with yield and quality. Therefore, in this study, those morphological traits were used as an investigation item to study drought tolerance.

Djulis (*C. formosana* Koidz.) is an endemic and herbaceous annual plant in Taiwan, which is a close relative of *C. quinoa* and a traditional food crop cultivated by the indigenous people. The plant grows vertically up to >2 m. Its seeds resemble the grains of the grass family; therefore, they are also called pseudocereal crops [14]. *C. formosana* is rich in dietary fiber, starch, and essential amino acids. Owing to its remarkably high nutritional value, it is considered to be one of the important food crops of the future [15]. Few studies have been conducted on the growth of *C. formosana* under drought conditions. Therefore, the present study used *C. quinoa* as the control to compare the drought stress responses of *C. formosana* and *C. quinoa*, aiming to investigate the impact of drought on the growth and yield of *C. formosana* and discuss the possible mechanism of its drought tolerance.

## 2. Results

### 2.1. Measurement of Plant Height and Leaf Water Content

**Figure 1:**
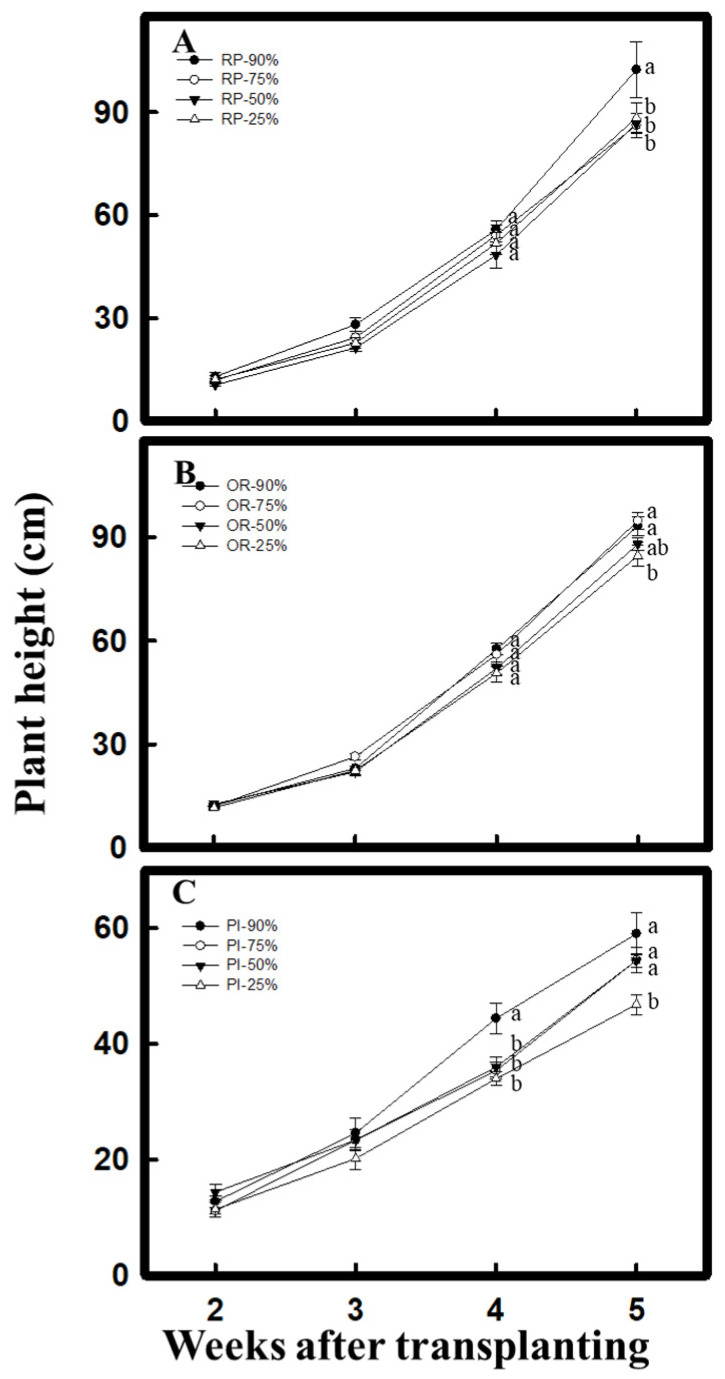
Effect of drought stress on plant height in *C. formosana* and *C. quinoa* during vegetative growth. From 2nd to 5th week after sowing, the plant heights of *C. formosana* RP variety (**A**), OR variety (**B**), and *C. quinoa* PI variety (**C**) were investigated. Bars show means ± SE. Values with the same letter are not significantly different by LSD (*p* < 0.05, *n* = 5).

**Figure 2:**
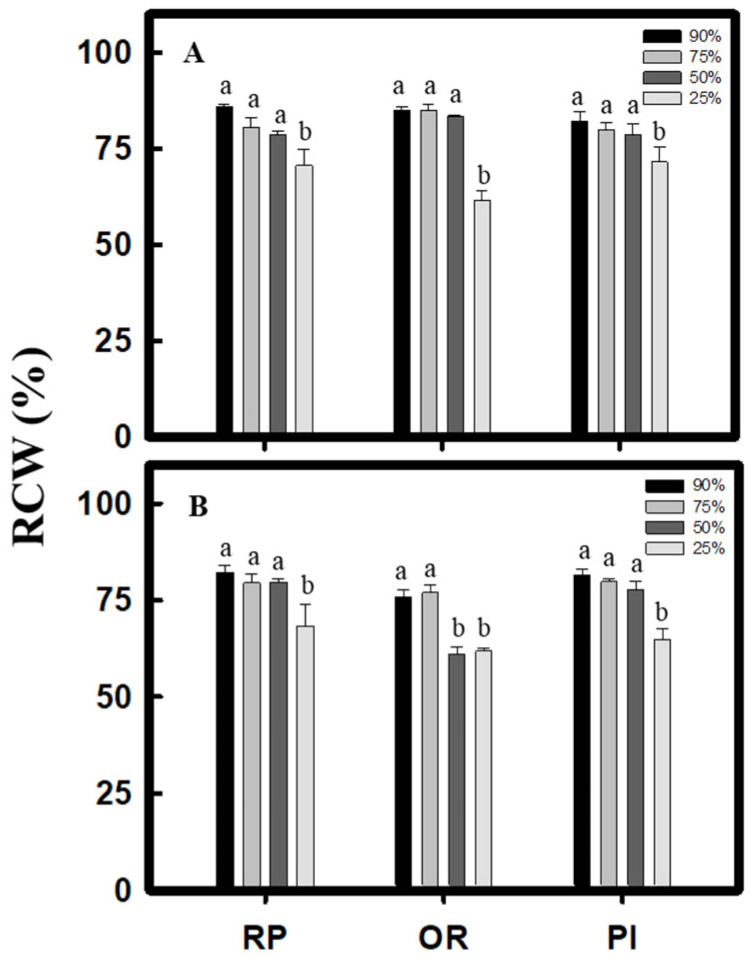
Effect of drought stress on relative water content (RWC) in *C. formosana* and *C. quinoa*. *C. formosana* RP variety, OR variety, and *C. quinoa* PI variety grow to vegetative stage (5 weeks after transplanting) (**A**) and reproductive stage (8 weeks after transplanting) (**B**); the water content was investigated, respectively. Bars show means ± SE. Values with the same letter are not significantly different in treatments between the same variety by LSD (*p* < 0.05, *n* = 4).

By the 5th week, after the 25% water content treatment, the plant height of all the test materials decreased significantly. For the 50% water content treatment, the OR variety of *C. formosana* and the PI variety of *C. quinoa* showed no significant change. The result indicated that both *C. formosana* and *C. quinoa* can adapt to the drought environment after experiencing drought treatment during vegetative growth (Figure 1). Moreover, after the 25% water content treatment, the leaf water content of *C. formosana* and *C. quinoa* during vegetative growth was 70.6% in RP, 61.6% in OR, and 71.5% in PI (Figure 2A). The leaf water content during reproductive growth was 68.3% in RP, 61.8% in OR, and 64.9% in PI (Figure 2B). The above results showed that under the 25% water content treatment, although the leaf water content of *C. formosana* and *C. quinoa* decreased significantly during both vegetative and reproductive growth phases, all the values remained above 50%. Among all the varieties tested, the water content of the OR variety fluctuated the least between the growth phases.

### 2.2. Analysis of Plant Physiological Indices

**Figure 3:**
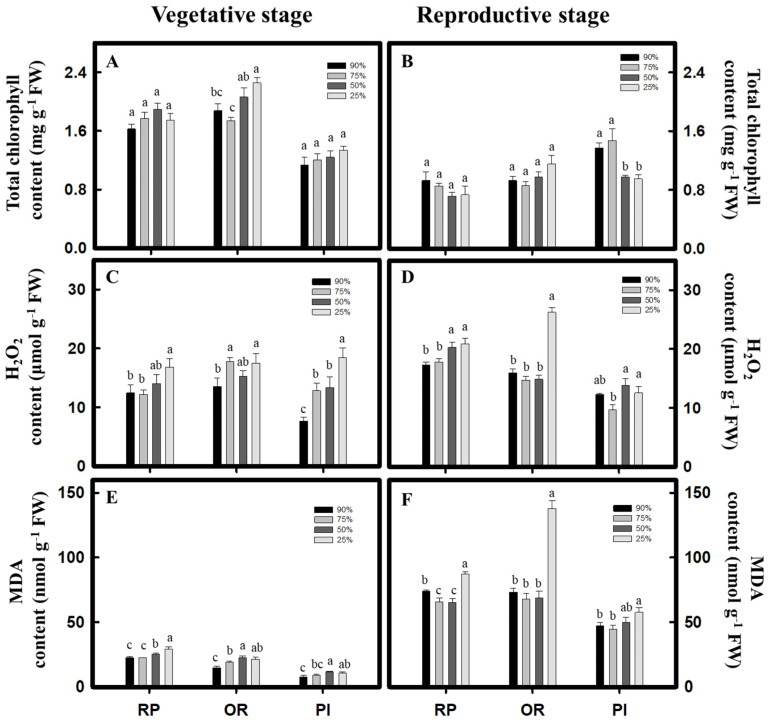
Effect of drought stress on physiological indicators in *C. formosana* and *C. quinoa*. *C. formosana* RP variety, OR variety, and *C. quinoa* PI variety during vegetative stage (5 weeks after transplanting) and reproductive stage (8 weeks after transplanting); the total chlorophyll content (**A**,**B**), H_2_O_2_ content (**C**,**D**), and MDA content (**E**,**F**) were investigated, respectively. Bars show means ± SE. Values with the same letter are not significantly different in treatments between the same variety by LSD (*p* < 0.05, *n* = 4).

*C. formosana* and *C. quinoa* showed no significant difference in the change of chlorophyll content between treatments during vegetative growth. The chlorophyll content of the OR variety of *C. formosana* increased significantly after the 25% water content treatment (2.2 mg g^−1^) (Figure 3A). During reproductive growth, the *C. formosana* varieties, RP and OR, showed no significant difference in chlorophyll contents between treatments. However, the PI variety of *C. quinoa* had significantly higher chlorophyll contents in the 90% and 75% water content treatments (1.3 mg g^−1^ and 1.4 mg g^−1^, respectively) than those in the 50% and 25% water content treatments (0.9 mg g^−1^ and 0.9 mg g^−1^, respectively) (Figure 3B). From the H_2_O_2_ content analysis, it was found that both *C. formosana* and *C. quinoa* raised the H_2_O_2_ content along with drought severity during vegetative growth. The PI variety of *C. quinoa* had the most remarkable increase (Figure 3C). The 25% water content treatment during reproductive growth led to significant increases of H_2_O_2_ content in all the varieties, and the OR variety of *C. formosana* showed the most remarkable increase (Figure 3D). Moreover, the RP variety of *C. formosana* had the most significant highest change in the malondialdehyde (MDA) content during vegetative growth (29.0 nmol g^−1^) under 25% water content treatment. The MDA contents of the *C. formosana* variety, OR, and *C. quinoa* variety, PI, were at the highest under 50% and 25% water content treatments (Figure 3E). During reproductive growth, the MDA contents of all the *C. formosana* and *C. quinoa* varieties increased along with the severity of drought treatment (Figure 3F), with the OR variety of *C. formosana* showing the most significant increase of MDA content.

### 2.3. Impact of Drought Treatment on Yield Components

*C. formosana* forms one primary spike without branching, whereas *C. quinoa* develops lateral spikes without a distinguishable primary spike. The two species also have different yield components.

For *C. formosana* and *C. quinoa*, both the wet and net weights of the aboveground parts tended to reduce under increasingly intense drought treatments. The same pattern also appeared in yield components such as inflorescence length, inflorescence weight, and grain weight. As the intensity of drought treatment increased, the spike number of both the *C. quinoa* varieties decreased. Except for the PI variety of *C. quinoa*, the inflorescence length of the *C. formosana* varieties, RP and OR, reduced significantly. For each variety, the weights of inflorescence and grain also decreased. However, there was no significant difference in the thousand grain weights between *C. formosana* and *C. quinoa* under various drought treatments (Table 1).

### 2.4. Analysis of Antioxidant Enzyme Activity

**Figure 4:**
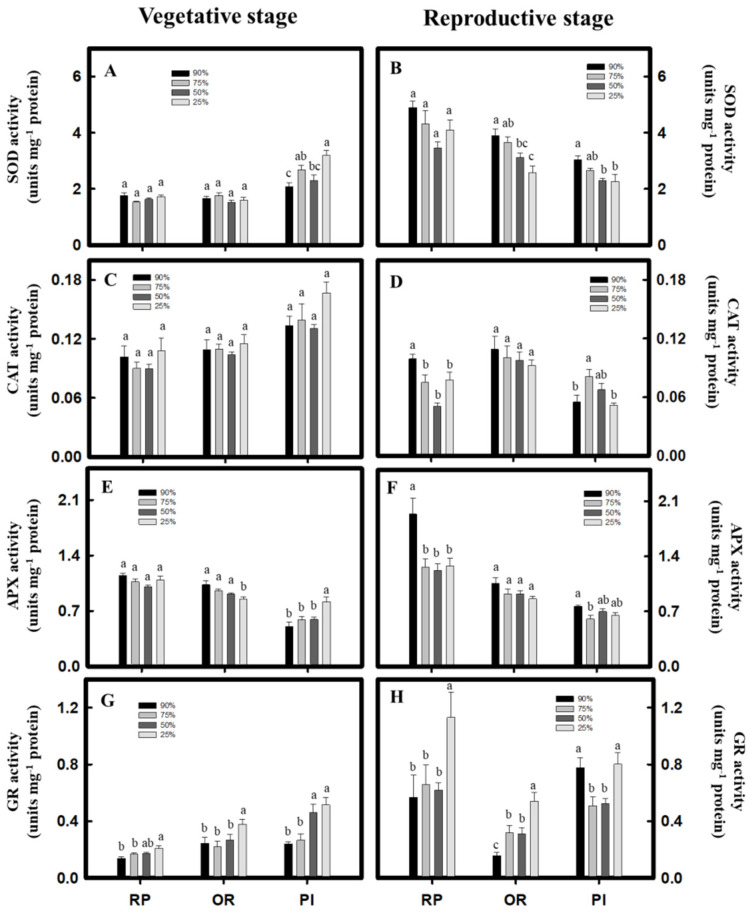
Effect of drought stress on antioxidant enzyme activities in *C. formosana* and *C. quinoa*. *C. formosana* RP variety, OR variety, and *C. quinoa* PI variety during vegetative stage (5 weeks after transplanting) and reproductive stage (8 weeks after transplanting); the SOD activity (**A**,**B**), CAT activity (**C**,**D**), APX activity (**E**,**F**), and GR activity (**G**,**H**) were investigated, respectively. Bars show means ± SE. Values with the same letter are not significantly different in treatments between the same variety by LSD (*p* < 0.05, *n* = 4).

During vegetative growth, *C. formosana* showed no significant difference in SOD activity across treatments. However, the SOD activity of the PI variety of *C. quinoa* increased along with the intensity of drought treatment (Figure 4A). For *C. formosana* and the PI variety of *C. quinoa*, CAT activities showed no significant differences among all the treatments (Figure 4C). In contrast, the APX activity showed a different pattern. For all the treatments, the RP variety of *C. formosana* showed no significant differences. Furthermore, the OR variety showed decreasing APX activity with the increasing intensity of the drought treatment, whereas the PI variety of *C. quinoa* displayed increasing activity (Figure 4E). Finally, in *C. formosana* and the PI variety of *C. quinoa*, the GR activity increased with the increasing intensity of the drought treatment (Figure 4G). During reproductive growth, the activities of SOD, CAT, and APX tended to decrease in the *C. formosana* varieties and the PI variety of *C. quinoa* as the drought intensity increased across the treatments (Figure 4B,D,F). The GR activity, however, increased significantly under the same circumstances (Figure 4H). In summary, the above results imply that the GR enzyme plays an important regulatory role under drought stress.

### 2.5. AsA and GSH Contents Analysis

The *C. formosana* varieties showed no significant changes in AsA content, dehydroascorbic acid (DHA) content, or AsA/DHA ratio across the treatments during either vegetative or reproductive growth. Only the OR variety displayed a trend of increase in AsA/DHA ratio during reproductive growth. During vegetative growth, the AsA content, DHA content, and AsA/DHA ratio of the PI variety of *C. quinoa* reached the highest level under the 25% water content treatment. During reproductive growth, the AsA/DHA ratio differed significantly at 25% water content (Table 2). With respect to the change of GSH content, during vegetative growth, the *C. formosana* varieties showed no significant change in the GSH content after the drought treatment, whereas their oxidized GSH (GSSG) content tended to increase. Therefore, the GSH/GSSG ratio dropped while the intensity of the drought increased. The GSH/GSSG ratio of the PI variety of *C. quinoa* showed a similar pattern. During reproductive growth, the GSH/GSSG ratio of the *C. formosana* varieties did not vary significantly across different drought treatments, whereas the GSH/GSSG ratio of the PI variety of *C. quinoa* showed significant differences (Table 2). The above results suggest that under drought treatments, the PI variety of *C. quinoa* experienced more increase in the AsA/DHA ratio and more decrease in the GSH/GSSG ratio than the *C. formosana* varieties.

### 2.6. Analysis of Proline and Carbohydrate Contents

**Figure 5:**
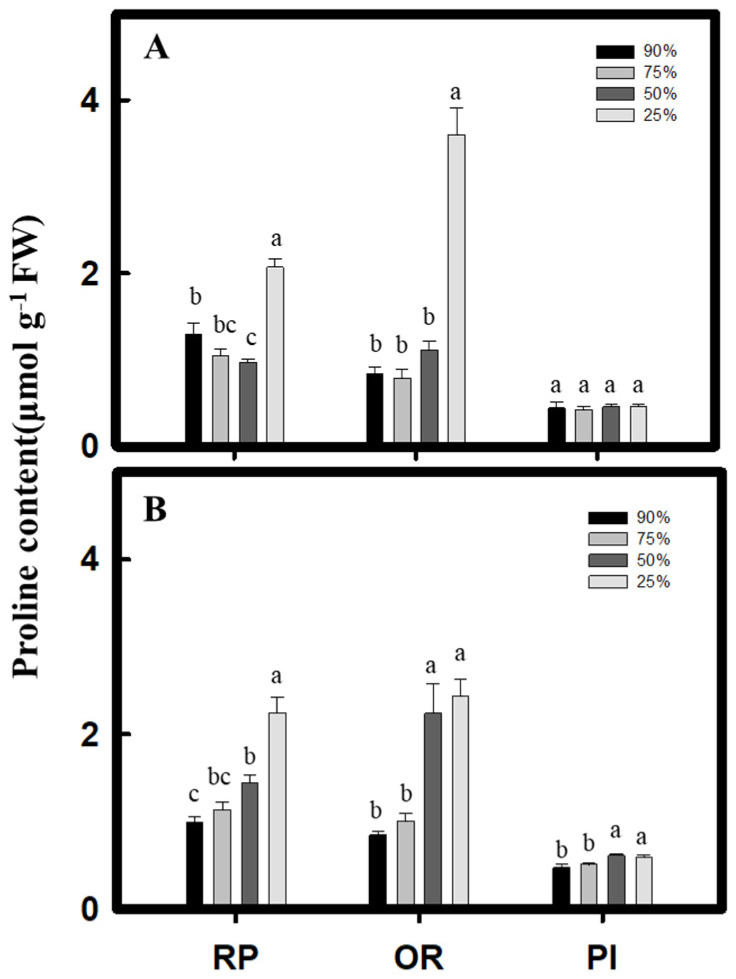
Effect of drought stress on proline content in *C. formosana* and *C. quinoa*. *C. formosana* RP variety, OR variety, and *C. quinoa* PI variety grow to vegetative stage (**A**) and reproductive stage (**B**); the proline content was investigated, respectively. Bars show means ± SE. Values with the same letter are not significantly different in treatments between the same variety by LSD (*p* < 0.05, *n* = 4).

**Figure 6:**
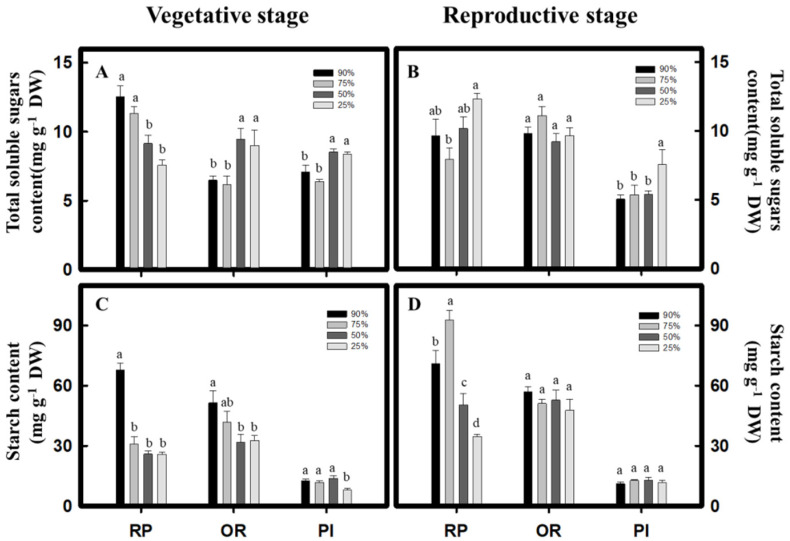
Effect of drought stress on total soluble sugars and starch content in *C. formosana* and *C. quinoa*. *C. formosana* RP variety, OR variety, and *C. quinoa* PI variety grow to vegetative stage and reproductive stage; the total soluble sugars (**A**,**B**) and starch content (**C**,**D**) were investigated, respectively. Bars show means ± SE. Values with the same letter are not significantly different in treatments between the same variety by LSD (*p* < 0.05, *n* = 4).

During vegetative growth, the *C. formosana* varieties, RP and OR, both had their significantly highest proline content under the 25% water content treatment, where it increased by 0.6- and 3.3-fold, respectively, compared to that under the 90% water content treatment. However, the PI variety of *C. quinoa* showed no significant difference in the proline content across the treatments (Figure 5A). During reproductive growth, the *C. formosana* varieties, RP and OR, and the *C. quinoa* variety, PI, reached their highest proline content under the 25% water content treatment, where it increased by 1.2-, 1.8-, and 0.3-fold, respectively, compared to that under the 90% water content treatment (Figure 5B). With regard to the total soluble sugar content, the *C. formosana* variety, OR, and the *C. quinoa* variety, PI, at vegetative growth showed a significant increase after the drought treatment. However, only the PI variety of *C. quinoa* showed a significant increase in total soluble sugar content during reproductive growth (Figure 6A,B). In contrast, during vegetative growth, the starch content of the *C. formosana* varieties, RP and OR, and the *C. quinoa* variety, PI, dropped to the lowest level under the 25% water content treatment. During reproductive growth, on the other hand, only the RP variety of *C. formosana* showed a decreasing starch content as the drought intensity increased (Figure 6C,D). The above results suggest that drought treatments induce the accumulation of osmosis-regulating substances. *C. formosana* mainly accumulates proline, whereas the PI variety of *C. quinoa* mainly increases the total soluble sugar content.

## 3. Discussion

Stress induces ROS generation in plants. Thus, plants have developed a defense mechanism to alleviate ROS damage to the cells and stabilize the cellular redox status. The defense mechanism consists of antioxidant enzymes, such as SOD, CAT, APX, and GR, as well as antioxidants, such as AsA and GSH [4,16]. The *C. quinoa* variety, Real Blanca, showed no significant difference in CAT activity between the drought treatment and control groups, but APX activity increased significantly under drought treatment [17]. In addition, another study has shown that compared with the control group (28% SVWC), the SOD and APX activities of *C. quinoa* under drought conditions (14% SVWC) increased significantly [16]. In the present study, the PI variety of *C. quinoa* showed significantly increased SOD and APX activities at vegetative growth after the 25% water content treatment (Figure 4A,E). This result is consistent with the conclusion of the study mentioned above. APX is one of the important enzymes regulating the equilibrium of the AsA/DHA ratio. The drought-tolerant tomato (*Solanum lycopersicum* L.) variety, Zarina, can maintain a high AsA/DHA ratio to reduce ROS generation [18]. In the drought environment, the drought-tolerant wheat variety, C306, has a higher AsA/DHA ratio than that of the drought-sensitive variety, Moti [19]. In this study, *C. formosana* and *C. quinoa* could still maintain relatively high AsA/DHA ratios after the 25% water content treatment, with the AsA/DHA ratio of the PI variety of *C. quinoa* being higher than that of the *C. formosana* varieties (Table 2).

Besides APX, the GR activity increased significantly in *C. formosana* and *C. quinoa* under 25% water content treatment (Figure 4G,H), but the GSH/GSSG ratio decreased (Table 1). A study on barley has demonstrated that GSH can effectively eliminate the H_2_O_2_ generated by photorespiration [20]. Under drought conditions, wheat (*Triticum aestivum* L.) reduces the GSH/GSSG ratio, and the drought-tolerant varieties may raise GR activity [20]. Furthermore, in a study on mung bean (*Vigna radiata* L.), drought treatment reduced the GSH/GSSG ratio while promoting GR activity [21]. GR is the last enzyme in ascorbic acid/glutathione cycle. Therefore, GR can maintain GSH content, alleviate oxidative stress damage, and maintain cell integrity under drought stress [22]. Cotton GR activity increased significantly during the recovery period after drought treatment, and the authors strongly suggested that drought could result in acclimation to greater water deficit [23]. Quinoa was found to significantly increase SOD and GR activities during drought treatment, which reduced the damage from oxidative stress [24]. The above results suggest that the AsA–GSH cycle in *C. formosana* and *C. quinoa* under drought stress could immediately reduce ROS generation, and the GR enzyme plays an essential regulatory role in the process.

In addition to increasing antioxidation capacity, crops under drought stress can also reduce cell osmotic potential by the regulation of osmosis to stabilize water absorption [4]. Proline can bind to proteins under drought stress to protect proteins from dehydration-induced denaturation, and it also stabilizes cell redox status [5,25]. Therefore, proline accumulates quickly under stress and acts as an important osmosis-regulating substance in plants. The study of González et al. (2009) [10] shows that the proline content of *C. quinoa* variety, Sajama, increased by 21% when subjected to a soil water potential of −0.20 MPa compared to that of the control group (soil water potential −0.05 MPa). The study of Sadak et al. (2019) [26] also indicated that the proline content of *C. quinoa* increased significantly under insufficient irrigation. In the present study, under the 25% water content treatment, the proline content of *C. formosana* and *C. quinoa* increased by 0.6- and 3.3-fold (for the RP and OR varieties, respectively) during vegetative growth and by 1.2-, 1.8-, and 0.3-fold (for the RP, OR, and PI varieties, respectively) during reproductive growth (Figure 5), indicating that the proline content in *C. formosana* increased more significantly than in *C. quinoa* under drought conditions to alleviate the damage caused by stress.

In addition to proline, the total soluble sugars can also regulate cell osmotic potential under drought stress to protect cell membrane structure while providing energy and carbon sources that are essential for plant growth and development [4,7]. The drought-tolerant rice variety, Zayandeh-Rood, has a high total soluble sugar content under drought stress [27]. The *C. quinoa* variety, Sajama, also increases total soluble sugar content under drought stress [10]. Under the drought treatments of this study, among the *C. formosana* varieties tested, only the OR variety accumulated total soluble sugars during vegetative growth (Figure 6A); however, the PI variety of *C. quinoa* could accumulate total soluble sugars during both vegetative and reproductive growth phases (Figure 6A,B). According to the above results, the *C. formosana* varieties, RP and OR, use proline as the primary osmosis-regulating substance under drought stress, whereas the *C. quinoa* variety, PI, primarily uses total soluble sugars.

Drought lowers the photosynthetic rate, reduces dry matter accumulation, and disrupts carbohydrate metabolism balance, which causes decreased spike numbers and affects grain filling, resulting in reduced yield [4,28]. In the study by Zhou et al. (2017) [29], the starch content of three tomato varieties decreased significantly under drought stress. In contrast, the *C. quinoa* variety, Sajama, showed no significant difference in leaf starch content between the drought treatment group (soil water potential at −0.20 MPa) and the control group (soil water potential at −0.05 MPa) [10]. In the present study, the *C. formosana* variety, RP, under drought treatment had significantly reduced starch content during both vegetative and reproductive growth phases. However, in the *C. formosana* variety, OR, and the *C. quinoa* variety, PI, the starch content only decreased significantly during vegetative growth (Figure 6C,D). A significant decrease in starch content indirectly reduces the aboveground dry and wet weights (Table 1), thereby affecting the yield components. Fischer et al. (2013) [30] demonstrated that, compared with the control group (with 95% field capacity), the yield of *C. quinoa* (with 20% field capacity) decreased by 38% in the Regalona variety, 35% in the B080 variety, and 17% in the AG2010 variety. In the present study, the spike number of both the *C. quinoa* varieties decreased with decreasing water content. Meanwhile, the inflorescence length and weight of *C. formosana* and *C. quinoa* reached the lowest values under the 25% water content treatment. However, no significant difference in thousand grain weight was observed (Table 1). The study of Al-Naggar et al. (2017) [31] indicates that the drought-tolerant *C. quinoa* variety, CICA-17, could maintain a high thousand grain weight even under drought conditions. The conclusion suggests that *C. formosana* and *C. quinoa* have relatively good drought tolerance and can maintain stable thousand grain weights under drought treatments.

Under drought stress, both *C. formosana* and *C. quinoa* increase their ability for antioxidation and content of osmosis-regulating substances to promote drought tolerance. Consequently, under the 25% water content treatment, although the plant height reduced, the change was not significant (Figure 1), and the leaf water content remained above 50% (Figure 2). Moreover, the chlorophyll content did not decrease significantly during vegetative growth (Figure 3A). Furthermore, on performing the experiments, we discovered that after 25% water content treatment, the OR variety of *C. formosana* would increase its total soluble sugar content (Figure 6A) with a fast accumulation of proline during vegetative growth, while raising the H_2_O_2_ and MDA contents significantly during reproductive growth (Figure 3D,F). The data indicate that the OR and RP varieties have similar drought tolerance to *C. quinoa*. However, OR varieties have a sensitive ability to accept water loss signal.

## 4. Materials and Methods

### 4.1. Plant Material Preparation and Drought Treatments

*C. formosana* seeds were provided by the lab of crop production and physiology (Department of Plant Industry, National Pingtung University of Science and Technology, Pintung, Taiwan); *C. quinoa* seeds were provided by National Plant Genetic Resources Center (Taichung, Taiwan).

The seeds with two *C. formosana* varieties, the red variety (RP) and the yellow variety (OR), and *C. quinoa* varieties, PI478414 (PI), were chosen as the test materials for the experiments. The seeds with varieties were planted in the soil: peat soil: pearlite = 3:2:1 medium. After growing in the greenhouse for four weeks, the seedlings were transplanted into plastic pots (diameter 29 cm, height 22 cm). Drought treatments were applied two weeks after the final transplantation. The drought treatment was to simulate the watering situation of the field and defined the field capacity when the potted watering reached the saturation point (32% VWC) in the experiment.

**Figure 7:**
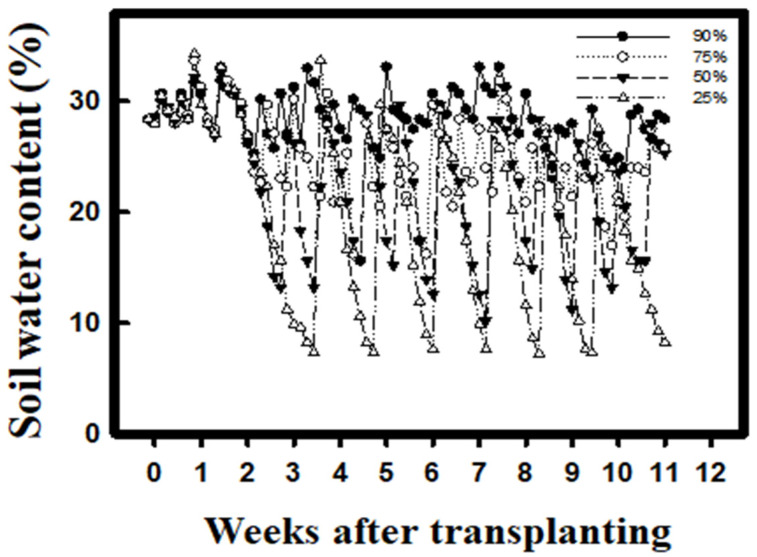
Soil water content change during growth season.

The VWC of the growing medium was monitored by WatchDog 1000 Series Micro Stations (Spectrum Technologies) during the experiments. The field capacity was set at 32% VWC. The intensity of drought treatments was as follows: 90% (28.5% VWC), 75% (24% VWC), 50% (16% VWC), and 25% (8% VWC). Each treatment included nine replicates. When the water content dropped below the treatment level, the pots were irrigated thoroughly to the field capacity (32% VWC) (Figure 7). Biochemical analysis was conducted at the fifth week (vegetative growth period) and eighth week (reproductive growth period, flowering stage) after transplanting. The analysis samples were selected from the 4th to 5th fully expanded leaves from the top bud.

### 4.2. Plant Height and Yield Survey

Plant heights were measured two weeks after final planting, and measurements were carried out once a week. The survey was concluded when 50% of the plants reached the heading stage. The yield component survey was conducted after *C. formosana* or *C. quinoa* had ripened. The survey items included spike number, inflorescence length, inflorescence weight, aboveground wet weight, aboveground dry weight, grain weight per plant, and thousand grain weight.

### 4.3. Analysis of Leaf Water Content and Physiological Parameters

For determining leaf water content, first, the fully unfolded leaves were collected to measure the wet weight. The leaves were then submerged in distilled water at room temperature for 24 h in the dark. The leaves were then blotted to remove the water from the leaf surfaces before weighing them to obtain the full turgor weight. Next, the leaves were dried at 70 °C for 72 h before measuring the dry weight. The formula for calculating the relative water content (RWC) is—RWC (%) = [(wet weight − dry weight)/(full turgor weight − dry weight)] ×100 [32]. Chlorophyll assay: the leaf samples were mixed with sodium phosphate buffer (50 mM, pH 6.8) and ground in an ice bath. The supernatant was mixed with 95% ethanol and kept in the dark for 30 min before being centrifuged at 1000× *g* under 4 °C for 15 min. The absorbance at wavelengths 665 and 649 nm was measured using a spectrophotometer (Hitachi, U-2900) [33]. Hydrogen peroxide (H_2_O_2_) assay: leaf samples were mixed with sodium phosphate buffer (50 mM, pH 6.8, containing 1 mM hydroxylamine) and ground in an ice bath. The ground sample was centrifuged at 6000× *g* under 4 °C for 25 min. The supernatant was mixed with titanium chloride (0.1% *v*/*v*, dissolved in 20% (*v*/*v*) H_2_SO_4_) and centrifuged at 6000× *g* at room temperature for 15 min. The absorbance was determined at a wavelength of 410 nm [34]. MDA assay: the leaf samples were ground in trichloroacetic acid (TCA, 5% *w*/*v*) before being centrifuged at 10,000× *g* under 20 °C for 5 min. The supernatant was mixed with thiobarbituric acid (0.5% *w*/*v*, containing 20% *w*/*v* TCA) and placed in a water bath at 95 °C for 30 min before centrifuging at 3000× *g* under room temperature for 10 min. The absorbance was determined at wavelengths of 532 and 600 nm [35]. The chlorophyll and H_2_O_2_ values were obtained directly after analyzing the plant leaves, so the unit was expressed in fresh weight.

### 4.4. Analysis of Proline and Carbohydrate Content

Proline assay: the sample was ground in 5 mL sulfosalicylic acid (3%, *w*/*v*) before centrifuging it at 5000× *g* under room temperature for 20 min. One milliliter supernatant was mixed with 1 mL ninhydrin and acetic acid. The mixture was kept in a 100 °C water bath and allowed to react for 60 min. Further, 4 mL toluene was added, followed by shaking for 15 s. The final mixture was allowed to stand for 10 min. The absorbance was determined at a wavelength of 520 nm [36]. The carbohydrate content was determined by the Dubois method [37].

### 4.5. Antioxidant Analysis

AsA and DHA assays were based on the reaction in which AsA reduces Fe^3+^ to Fe^2+^ in the presence of 5% TCA. Fe^2+^ was then mixed with bipyridyl to produce a red compound that can be measured at a wavelength of 525 nm [38]. GSH and GSSG contents were determined as described by Smith (1985) [39]. The assay was based on the 5,5′-dithio-bis-nitrobenzoic acid (DTNB)–GR reaction cycle, which produces a yellow compound that can be measured at a wavelength of 410 nm.

### 4.6. Antioxidant Enzyme Activity Analysis

The leaf samples were mixed with 3 mL sodium phosphate buffer (50 mM, pH 7.4) and ground in an ice bath before being centrifuged at 15,000× *g* under 4 °C for 30 min. The supernatant was mixed with triethanolamine–diethanolamine buffer (100 mM, pH 7.4), EDTA/MnCl_2_ (100 mM/50 mM, pH 7.4), 2-mercaptoethanol (10 mM), and NADH (7.5 mM). The SOD activity was determined by a spectrophotometer (Hitachi, U-2900) at 340 nm. In this study, one unit of SOD was defined as the enzyme activity that inhibits 50% of the NADH oxidation rate in blank samples [40].

The leaf samples were mixed with 4 mL sodium phosphate buffer (50 mM, pH 6.8) and ground in an ice bath before being centrifuged at 12,000× *g* under 4 °C for 20 min. The supernatant was used for determining APX, CAT, and GR activities. APX activity assay: the supernatant was mixed with potassium phosphate buffer (150 mM, pH 7.0), AsA (1.5 mM), ethylenediaminetetraacetic acid (0.75 mM), and H_2_O_2_ (6 mM). Then, the APX activity was measured at 290 nm. One unit of APX was defined as the amount of APX required to decompose 1 mole of AsA per min [41]. CAT activity assay: the supernatant was mixed with sodium phosphate buffer (100 mM, pH 7.0) and H_2_O_2_ (1 M), and the CAT activity was measured at 240 nm. One unit of CAT activity was defined as the amount of CAT required to decompose 1 mole of H_2_O_2_ per min [42]. GR activity assay: the supernatant was mixed with Tris-HCl buffer (150 mM, pH 7.5), MgCl_2_ (30 mM), GSSG (3 mM), and NADPH (0.45 mM), and the GR activity was measured at 340 nm. One unit of GR was defined as the amount of enzyme required to decrease 1 absorbance per min at 340 nm [43].

### 4.7. Statistical Analysis

The experiments adopted a completely randomized design. The least significant difference test was conducted using the statistics software SAS 9.4 (SAS Inc., Cary, NC, USA) to compare the difference between treatments (*p* ≤ 0.05).

## 5. Conclusions

**Figure 8:**
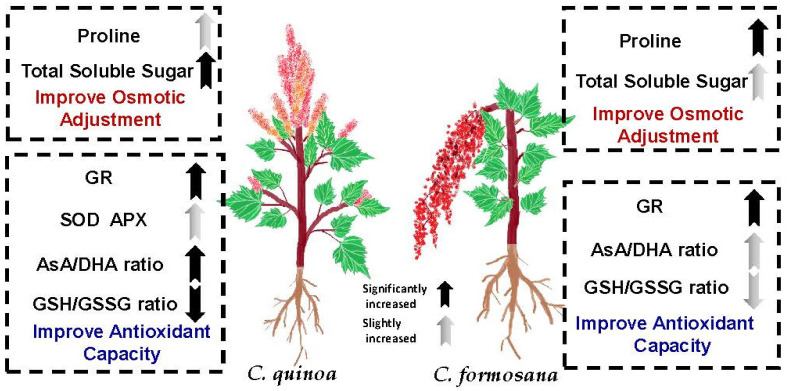
Different physiological mechanisms of drought tolerance between *C. formosana* and *C. quinoa*.

In summary, the above results show that *C. formosana* and *C. quinoa* have similar drought tolerance in this study. Therefore, *C. formosana* and *C. quinoa* use different drought tolerance mechanisms to improve drought tolerance (Figure 8). However, the activities of *C. formosana* and *C. quinoa* GR increased significantly under drought stress; it has been shown that GR has an important regulating effect when water is inadequate. In the future, in the breeding process of *C. formosana* and *C. quinoa,* GR activity can be used as an indicator of drought tolerance.

## Figures and Tables

**Table 1:** Effect of drought stress on growth and yield components in *C. formosana* and *C. quinoa*.

Variety	Treatment	Spike Number	Inflorescence Length (cm)	Inflorescence Weight (g)	Shoot Fresh Weight (g)	Shoot Dry Weight (g)	Grain Weight (g)	1000 Seeds Weight (g)
RP	90%	ND	60.0 ± 2.3a	64.8 ± 4.1a	132.0 ± 9.6a	44.5 ± 3.1a	25.1 ± 2.6a	1.37 ± 0.03a
	75%	ND	62.3 ± 1.9a	53.2 ± 3.1b	98.3 ± 4.7bc	33.1 ± 1.6b	19.0 ± 1.5b	1.26 ± 0.04a
	50%	ND	61.5 ± 1.3a	58.4 ± 2.2ab	116.8 ± 8.5ab	42.8 ± 4.9ab	20.4 ± 1.5ab	1.34 ± 0.10a
	25%	ND	53.2 ± 1.4b	40.2 ± 1.9c	85.1 ± 6.5c	32.6 ± 3.1b	13.4 ± 1.3c	1.35 ± 0.04a
OR	90%	ND	65.5 ± 1.0a	62.3 ± 2.6a	106.5 ± 5.9a	40.8 ± 2.2a	22.9 ± 1.3a	1.48 ± 0.06a
	75%	ND	62.6 ± 3.3a	66.1 ± 7.0a	116.9 ± 11.4a	46.6 ± 3.3a	23.4 ± 3.6a	1.57 ± 0.02a
	50%	ND	46.5 ± 1.1b	34.1 ± 2.1b	75.8 ± 3.6b	27.9 ± 2.2b	14.7 ± 1.3b	1.48 ± 0.03a
	25%	ND	46.5 ± 1.5b	42.0 ± 2.3b	79.4 ± 3.6b	31.7 ± 1.5b	16.1 ± 1.5b	1.55 ± 0.06a
PI	90%	20.2 ± 0.9a	27.4 ± 2.2a	92.2 ± 4.8a	119.5 ± 4.5a	28.5 ± 3.2a	9.3 ± 2.2a	1.61 ± 0.07a
	75%	18.7 ± 1.3ab	29.6 ± 2.0a	94.7 ± 10.3a	131.3 ± 14.4a	32.5 ± 3.1a	11.3 ± 1.2a	1.73 ± 0.03a
	50%	16.7 ± 1.1b	28.1 ± 0.7a	79.2 ± 7.3a	110.3 ± 9.2a	24.4 ± 3.2ab	7.9 ± 1.3ab	1.63 ± 0.05a
	25%	12.3 ± 0.3b	29.6 ± 1.0a	52.5 ± 6.5b	68.3 ± 8.4b	20.0 ± 1.1b	4.5 ± 0.7b	1.57 ± 0.03a

Values are means (±SE) of four replicates, for each treatment; different letters represent significant differences among the same variety according to LSD at *p* < 0.05 (*n* = 4). ND = No data.

**Table 2:** Effect of drought stress on antioxidant content in *C. formosana* and *C. quinoa*.

Antioxidant	Variety	Treatment							
		Vegetative Stage				Reproductive Stage			
		90%	75%	50%	25%	90%	75%	50%	25%
ASA content (μmol g^−1^ FW)	RP	9.47 ± 0.65a	8.33 ± 0.42a	9.67 ± 0.96a	9.19 ± 0.71a	10.07 ± 0.86a	12.11 ± 0.83a	10.85 ± 0.56a	11.46 ± 0.64a
	OR	9.73 ± 0.40a	10.33 ± 0.21a	10.94 ± 0.60a	10.72 ± 0.14a	14.37 ± 0.63a	13.88 ± 1.30a	13.20 ± 0.43a	15.24 ± 1.14a
	PI	1.79 ± 0.56b	2.26 ± 0.13b	2.46 ± 0.07b	4.08 ± 0.27a	5.33 ± 0.80a	4.21 ± 0.45a	4.18 ± 0.22a	5.88 ± 0.79a
DHA content (μmol g^−1^ FW)	RP	6.44 ± 0.32a	5.51 ± 0.57a	6.03 ± 0.24a	6.23 ± 0.29a	6.85 ± 0.25ab	7.46 ± 0.28a	6.19 ± 0.17b	6.83 ± 0.25ab
	OR	6.43 ± 0.38a	6.97 ± 0.32a	6.40 ± 0.27a	6.29 ± 0.21a	7.17 ± 0.22a	8.02 ± 0.35a	7.45 ± 0.17a	7.25 ± 0.39a
	PI	2.45 ± 0.73b	3.20 ± 0.12b	3.27 ± 0.11b	4.06 ± 0.16a	5.32 ± 0.58a	4.66 ± 0.34a	4.56 ± 0.28a	4.95 ± 0.16a
ASA/DHA ratio	RP	1.5 ± 0.2a	1.6 ± 0.1a	1.6 ± 0.1a	1.5 ± 0.1a	1.5 ± 0.1a	1.5 ± 0.1a	1.8 ± 0.1a	1.7 ± 0.1a
	OR	1.5 ± 0.1b	1.5 ± 0.1b	1.6 ± 0.1ab	1.7 ± 0.1a	2.0 ± 0.1a	1.7 ± 0.0b	1.8 ± 0.0b	2.1 ± 0.1a
	PI	0.7 ± 0.1b	0.7 ± 0.1b	0.8 ± 0.1b	1.0 ± 0.1a	1.0 ± 0.1b	0.9 ± 0.1b	0.9 ± 0.1b	1.2 ± 0.1a
GSH content (nmol g^−1^ FW)	RP	7.85 ± 0.91a	6.18 ± 0.72a	6.73 ± 0.85a	6.43 ± 0.69a	3.78 ± 0.38b	7.99 ± 0.93a	4.44 ± 0.47b	5.40 ± 0.46b
	OR	9.98 ± 0.45b	13.61 ± 0.83a	9.77 ± 0.80b	10.30 ± 0.55b	10.29 ± 0.97a	4.72 ± 0.59b	5.40 ± 0.70b	8.51 ± 1.00a
	PI	3.35 ± 0.30a	3.05 ± 0.22a	3.65 ± 0.44a	2.90 ± 0.31a	3.30 ± 0.30a	3.44 ± 0.30a	2.98 ± 0.53a	3.18 ± 0.43a
GSSG content (nmol g^−1^ FW)	RP	4.61 ± 0.34b	5.75 ± 0.27b	5.42 ± 0.51b	7.28 ± 0.53a	13.35 ± 0.60c	22.85 ± 1.53a	17.65 ± 0.53b	23.01 ± 1.39a
	OR	6.07 ± 0.20b	5.07 ± 0.36b	5.83 ± 0.45b	7.51 ± 0.35a	28.98 ± 1.65a	19.87 ± 1.82b	21.43 ± 1.66b	24.08 ± 1.22ab
	PI	1.61 ± 0.23a	1.83 ± 0.39a	1.70 ± 0.32a	2.34 ± 0.11a	2.37 ± 0.07b	2.48 ± 0.47b	2.48 ± 0.16b	4.06 ± 0.28a
GSH/GSS Gratio	RP	1.6 ± 0.1a	1.2 ± 0.1b	1.1 ± 0.1b	0.9 ± 0.1b	0.3 ± 0.1b	0.3 ± 0.1a	0.3 ± 0.1b	0.2 ± 0.1b
	OR	1.7 ± 0.1ab	1.8 ± 0.3a	1.6 ± 0.1ab	1.2 ± 0.1b	0.4 ± 0.1a	0.3 ± 0.1b	0.2 ± 0.1b	0.4 ± 0.1a
	PI	2.2 ± 0.2a	2.1 ± 0.3a	2.0 ± 0.3a	1.2 ± 0.1b	1.4 ± 0.1a	1.3 ± 0.2a	1.3 ± 0.1a	0.7 ± 0.1b

Values are means (± SE) of four replicates, for each treatment; different letters represent significant differences among the same variety according to LSD at *p* < 0.05 (*n* = 4).

## Data Availability

The data presented in this study are available on request from the corresponding author.
